# MiR-132 Suppresses the Migration and Invasion of Lung Cancer Cells *via* Targeting the EMT Regulator ZEB2

**DOI:** 10.1371/journal.pone.0091827

**Published:** 2014-03-13

**Authors:** Jiacong You, Yang Li, Nianzhen Fang, Bin Liu, Lingling Zu, Rui Chang, Xuebing Li, Qinghua Zhou

**Affiliations:** Tianjin Key Laboratory of Lung Cancer Metastasis and Tumor Microenvironment, Tianjin Lung Cancer Institute, Tianjin Medical University General Hospital, Tianjin, China; National Taiwan University, Taiwan

## Abstract

MicroRNAs (miRNAs) are small, non-coding RNAs which can function as oncogenes or tumor suppressor genes in human cancers. Emerging evidence reveals that deregulation of miRNAs contributes to the human non-small cell lung cancer (NSCLC). In the present study, we demonstrated that the expression levels of miR-132 were dramatically decreased in examined NSCLC cell lines and clinical NSCLC tissue samples. Then, we found that introduction of miR-132 significantly suppressed the migration and invasion of lung cancer cells in vitro, suggesting that miR-132 may be a novel tumor suppressor. Further studies indicated that the EMT-related transcription factor ZEB2 was one direct target genes of miR-132, evidenced by the direct binding of miR-132 with the 3′ untranslated region (3′ UTR) of ZEB2. Further, miR-132 could decrease the expression of ZEB2 at the levels of mRNA and protein. Notably, the EMT marker E-cadherin or vimentin, a downstream of ZEB2, was also down-regulated or up-regulated upon miR-132 treatment. Additionally, over-expressing or silencing ZEB2 was able to elevate or inhibit the migration and invasion of lung cancer cells, parallel to the effect of miR-132 on the lung cancer cells. Meanwhile, knockdown of ZEB2 reversed the enhanced migration and invasion mediated by anti-miR-132. These results indicate that miR-132 suppresses the migration and invasion of NSCLC cells through targeting ZEB2 involving the EMT process. Thus, our finding provides new insight into the mechanism of NSCLC progression. Therapeutically, miR-132 may serve as a potential target in the treatment of human lung cancer.

## Introduction

Lung cancer is one of the most common causes of cancer-related deaths worldwide, and majority of lung cancers are the non- small cell lung cancer (NSCLC), which comprises approximately 80% of all lung cancers [1]. Patients harboring NSCLC are frequently diagnosed as an advanced stage, suffering by metastatically or locally advanced diseases, making nearly 90% of lung cancer patients die of metastasis [2]. Although great efforts and progressions have been made in the study of the lung cancer in recent decades, the molecular mechanism of lung cancer metastasis remains elusive.

The microRNA (miRNA) is a class of small, non-coding RNAs with approximately 19–25 nucleotides. It negatively regulates gene expression at post-transcription level by interacting with the 3′ untranslated regions (3′- UTRs) of target mRNAs [3], [4]. MiRNAs are phylogenetically conserved and play crucial roles in a number of biological processes including development, differentiation, apoptosis, metabolism, immunity and tumor progress [5], [6]. Also, increasing evidence indicates microRNAs can modulate tumor initiation and progression and function in tumor cell invasion and metastasis [7], [8], [9], [10]. Previous studies have documented the roles of miR-132 in regulating the differentiation of dopamine neurons [11] and activating the endothelium to facilitate pathological angiogenesis [12]. In the tumorigenesis, it is reported that downregulation of miR-132 contributes to pancreatic cancer development [13]. However, the potential role of miR-132 in lung cancer progression has still not been documented.

ZEB2/SIP1 is a member of the deltaEF-1 family of two-handed zinc-finger factors and play vital roles in the development of a variety of cancers, such as gastric, ovarian, squamous and non-small cell lung carcinomas [14], [15]. ZEB2 specifically suppress the expression of E-cadherin through binding to CACCT(G) motif in the E-cadherin promoter during epithelial- mesenchymal transition (EMT) [16], [17]. Besides E-cadherin, other genes like plakophilin 2 and ZO-3 which involve epithelial cell-cell junctions are also repressed by ZEB2 [18]. Recently, ZEB2 is reported to transcriptionally up-regulate vimentin *via* cooperation with Sp1 during EMT [19].

In the present study, we sought to investigate the putative role of miR-132 in metastasis of NSCLC. We found that miR-132 is down-regulated in metastatic lung cancer cell lines and clinical tissue samples, suggesting that miR-132 might act as a tumor suppressor. We identified that the EMT regulator ZEB2 is one of direct target genes of miR-132. MiR-132 is able to inhibit EMT and metastasis of NSCLC cells through paralyzing the function of ZEB2.

## Materials and Methods

### Ethics Statement

The study was approved by the Ethics Committee of Tianjin Medical University, China, and written informed consents were obtained from all studied patients.

### Cell Lines and Clinical Specimens

The sub-cell lines, high- metastatic L9981 and low- metastatic NL9980, were isolated and established from a human lung large cell carcinoma cell line [20]. The high- metastatic 95D and low- metastatic 95C were sublines of human giant-cell lung carcinoma cell line [21]. The NSCLC cell line YTMLC-9 [22], [23] was established in our institute. These cell lines were cultured in RPMI-1640 medium supplemented with 10% calf serum (Invitrogen, USA), 100 IU/ml penicillin and 100 IU/ml streptomycin. The NSCLC cell line A549, purchased from the American Tissue Culture Collection (ATCC), cultured in DMEM medium supplemented with 10% fetal bovine serum, 100 U/mL penicillin and 100 U/mL streptomycin. These cell lines were grown at 37°C in a humidified atmosphere with 5% CO_2_. For the transfections, cells were grown to 70% confluence and transfected with plasmids using Lipofectamine2000 (Invitrogen) according to the manufacturer’s recommendation.

A total of 90 cases of NSCLC specimens were obtained from General Hospital of Tianjin Medical University. All 90 patients hadn’t received radiation therapy or chemotherapy prior to the surgery. Tissue samples for use were stored in liquid nitrogen. The TNM staging system of the UICC (1997) was used to classify the specimens and the survival times which were calculated from the operation day to death via the evaluation of recurrence and metastasis until the last follow-up date. The following-up of the surviving patients averaged 32.55 months and ranged from 1 to 96 months. The study has been approved by hospital ethical committee. Clinicopathological information of the patients about age, tumor size, histological type, differentiation, stage and lymph node metastasis was obtained from patient records, which were summarized in Table 1.

**Table 1:** The association of miR-132 with clinicopathological features of 90 patients with non-small cell lung cancer.

Factor		MiR-132 Expression		Z value	P value
	Median	25%	75%		
Age					
<60	0.0157	0.0079	0.0273		
≥60	0.0141	0.0092	0.0171	−0.796	0.426
Gender					
Man	0.0145	0.0097	0.0174		
Woman	0.0124	0.0045	0.0186	−0.465	0.642
Smoking history					
Smoker	0.1455	0.0097	0.0167		
Non smoker	0.0135	0.0070	0.0399	−0.245	0.806
Histology					
Sq	0.0144	0.0092	0.0173		
Ad	0.0146	0.0087	0.0271	−0.725	0.468
Stage					
I+II	0.0146	0.0087	0.0271		
III+IV	0.0144	0.0072	0.1738	−0.245	0.806
Lymph node status					
Negative	0.0600	0.0135	0.1219		
Positive	0.0141	0.0076	0.0162	−2.533	**0.011**

### Reverse-transcription PCR (RT-PCR), Quantitative Real-time Polymerase Chain Reaction (qRT-PCR) and Werstern Blot Assay

Total RNA extracted by the mirVana Kit (Applied Biosystems, CA) was reversely transcribed to cDNA with the stem-loop reverse transcription primer for miRNA detection. Reverse transcription of miR-132 and internal control U6 was performed using Reverse Transcriptase M-MLV (Takara, Japan). The qRT- PCR was performed using SYBR Premix Ex Taq (Takara) following the protocol using a preheated real-time instrument (Invitrogen). All primers were shown in Table S1. Three independent experiments were conducted to analyze relative gene expressions and each sample was tested in triplicate. Ct values were used to calculate the expression of RNA levels. The amount of target gene expression (2^−ΔΔCt^) was normalized using U6 reference.

### Plasmid Constructions

Genomic sequence of human miR-132, including ∼200 bp flanking sequence, was amplified from human genome, then inserted into the BamHI/EcoRI site of the pcDNA3.1 vector (Invitrogen), named as pcDNA3.1-miR-132. The full-length 3′untranslated region (3′UTR) of ZEB2 was amplified from human genomic DNA, and was cloned into the downstream of the firefly luciferase coding region of pMIR-GLOTM Luciferase vector (Promega, USA). The recombined vector was named as pMIR-ZEB2. Mutations of miR-132 binding sites were introduced by site-directed mutagenesis and the resulted vector was named pMIR-ZEB2-Mut. Primers used for the constructions were listed in Table S1. All the constructions were confirmed by sequencing.

### Antibodies and siRNAs

Primary antibodies used in Western blot were rabbit anti-ZEB2 (Santa Cruz, USA), anti-E-cadherin (Santa Cruz), anti-vimentin (Santa Cruz) and mouse anti-β-actin (Sigma, Aldrich, St. Louis, MO). β-actin was used for normalization. The small interfering RNAs (siRNA) targeting human ZEB2 mRNA, negative control siRNA (siControl), miR-132 inhibitor (anti-miR-132), and inhibitor negative control (anti-miR-NC) were purchased from Ruibobio (Guangzhou, China). All oligonucleotide sequences are listed in Table S1.

### Cell Migration and Invasion Assays

Wound healing assay was performed to analyze the cell migration as described previously [24]. Boyden chamber assays were used to examine cell invasion capability. Cells were transfected with 1 µg of pcDNA3.1 or pcDNA3.1-miR-132 vector. Sixteen hours later, transfected cells were trypsinized and resuspended. 1.0×10^5^ cells in 300 µl culture medium were placed into the upper chambers (Millipore). The lower chambers were filled with 500 µl complete medium with 10% FBS. After incubation for 12 h at 37°C, non-invading cells were removed from the top of the chamber with a cotton swab. The migratory cells on the lower surface of the inserts were fixed and stained with 0.1% crystal violet, and five random fields for each insert were counted at 100×magnifications.

### Luciferase Reporter Gene Assay

Adherent cells were seeded into 24-well plates and co-transfected with 200 ng of pMIR-ZEB2 or pMIR-ZEB2-Mut vector and 80 ng of pcDNA3.1-miR-132 vector or pcDNA 3.1 empty vectors, and the pRL-TK plasmid (Promega, Madison, WI) which is used as internal normalization. Cells were harvested after 36 hr and lysed using the lysis buffer (Promega). Luciferase reporter gene assay was implemented using the Dual-Luciferase Reporter Assay System (Promega) according to the manufacturer’s instructions. All experiments were performed at least three times.

### Statistical Analysis

Each experiment was repeated at least three times. Statistical significance was assessed by comparing mean values (± SD) using a Student’s *t-*test for independent groups and was assumed for *p*<0.05 (*) and *p*<0.01 (**).

## Results

### MiR-132 is Frequently Down-regulated in Highly Metastatic Lung Cancer Cells and Tissue Specimens

**Figure 1:**
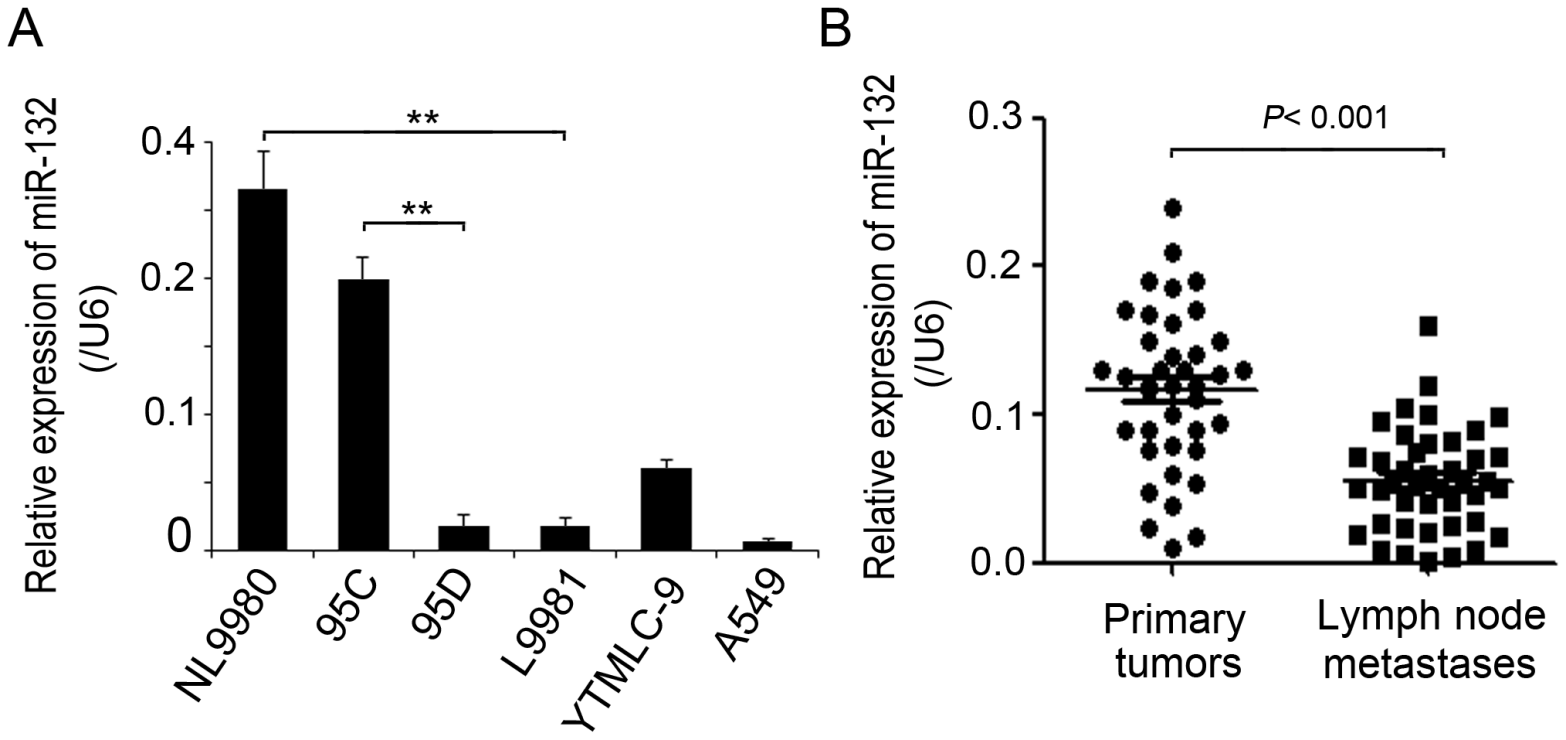
MiR-132 is frequently down-regulated in highly metastatic lung cancer cells and tissue specimens. (A) The relative mRNA levels of miR-132 were detected by qRT-PCR and normalized against an endogenous control (U6 RNA) in several lung cancer cell lines with distinct metastatic ability. Data are reported as mean ±SD for three independent experiments (**P<0.01, Student’s *t*- test). (B) qRT-PCR analysis of miR-132 expression in 45 pairs of primary NSCLC tissues and their corresponding lymph node metastases. MiR-132 expression in those two types of tissues was compared by way of Wilcoxon signed-rank test (***P<0.001, Student’s *t*- test).

We evaluated the expression of miR-132 by qRT-PCR in several NSCLC cell lines with distinct metastatic capacities. We found that miR-132 levels exhibited a varying pattern in these cell lines. Notably, the expression of miR-132 in highly metastatic L9981 and 95D cells was dramatically diminished, relative to that in the corresponding poorly metastatic NL9980 or 95C cell lines, respectively (Figure 1A). Further, we detected miR-132 expression in 45 paired clinical primary lung cancer tissue and metastatic lymph node cancer tissue samples. Compared to their primary counterparts, the lymph metastatic tissue samples harbored a significant lower miR-132 levels (Figure 1B, *P*<0.001, student’s *t*-test). Also, the analysis on clinicopathological features of patients with NSCLC showed that expression of miR-132 had a significant correlation with lymph node status (Table 1, *P* = 0.011). These data indicate that the reduced expression of miR-132 is a frequent event in highly metastatic NSCLC cells and tissues, which may be involved in the metastasis of human lung cancer cells.

### MiR-132 is Able to Inhibit the Migration and Invasion of NSCLC Cells in vitro

**Figure 2:**
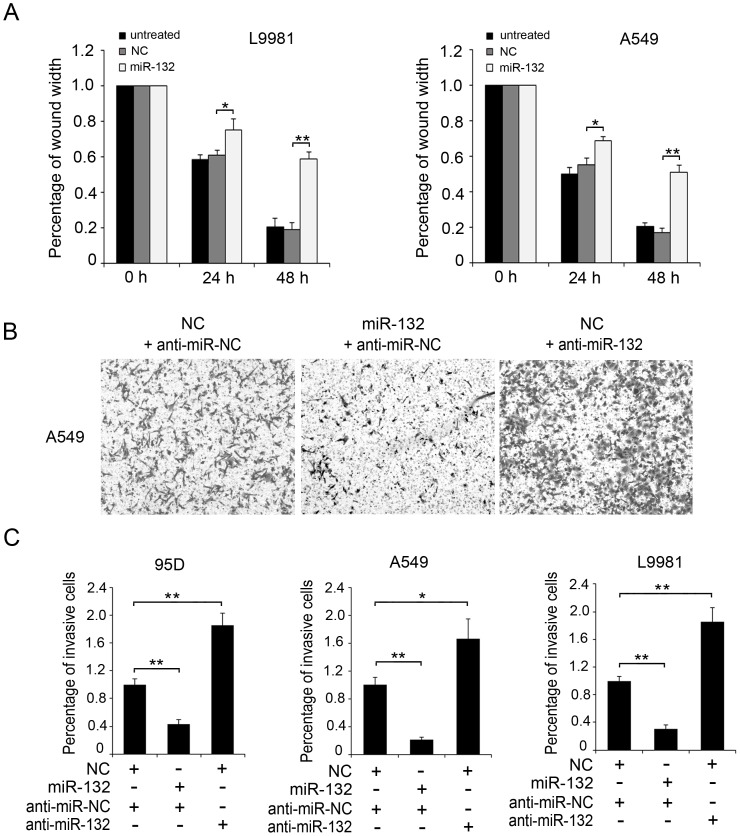
MiR-132 is able to inhibit the migration and invasion of NSCLC cells in vitro. (A) The wound healing or was used to detected the migration ability of L9981 and A549 cells, respectively. (B, C) Boyden chamber assay was employed to examine the invasion ability of 95D, L9981, and A549 cells, respectively. The results were from three independent experiments (*P<0.05, **P<0.01, Student’s *t*- test). The migratory cell number in each group was normalized to the control. Cells were transfected with pcDNA3.1 (NC) or pcDNA3.1-miR-132 (miR-132) constructs, and miR-132 inhibitor (anti-miR-132) or inhibitor control (anti-miR-NC). The migratory A549 cells in lower chambers from one experiment were shown in B.

Next, we tested the functional significance of miR-132 in NSCLC cells. Wound healing assay showed that the ectopic expression of miR-132 in L9981 or A549 lung cancer cells significantly inhibited cell migration, compared to the control group (Figure 2A). Additionally, we performed the Boyden chamber assay to investigate the effect of miR-132 on cell invasion. As shown in Figure 2B and 2C, when transfected with pcDNA3.1-miR-132 plasmids, the invasion ability of A549 cells exhibited an over-3 fold decrease, compared to the control group. However, the cells showed an increased invasion upon the treatment of miR-132 inhibitor (Figure 2B, 2C). In addition, parallel results were observed in 95D and L9981 cell lines (Figure 2C). Taken together, the data strongly suggest that miR-132 is able to suppress the migration and invasion of NSCLC cells *in vitro*.

### MiR-132 Directly Inhibits the Expression of ZEB2 through its 3′UTR and Regulates the EMT of NSCLC Cells

**Figure 3:**
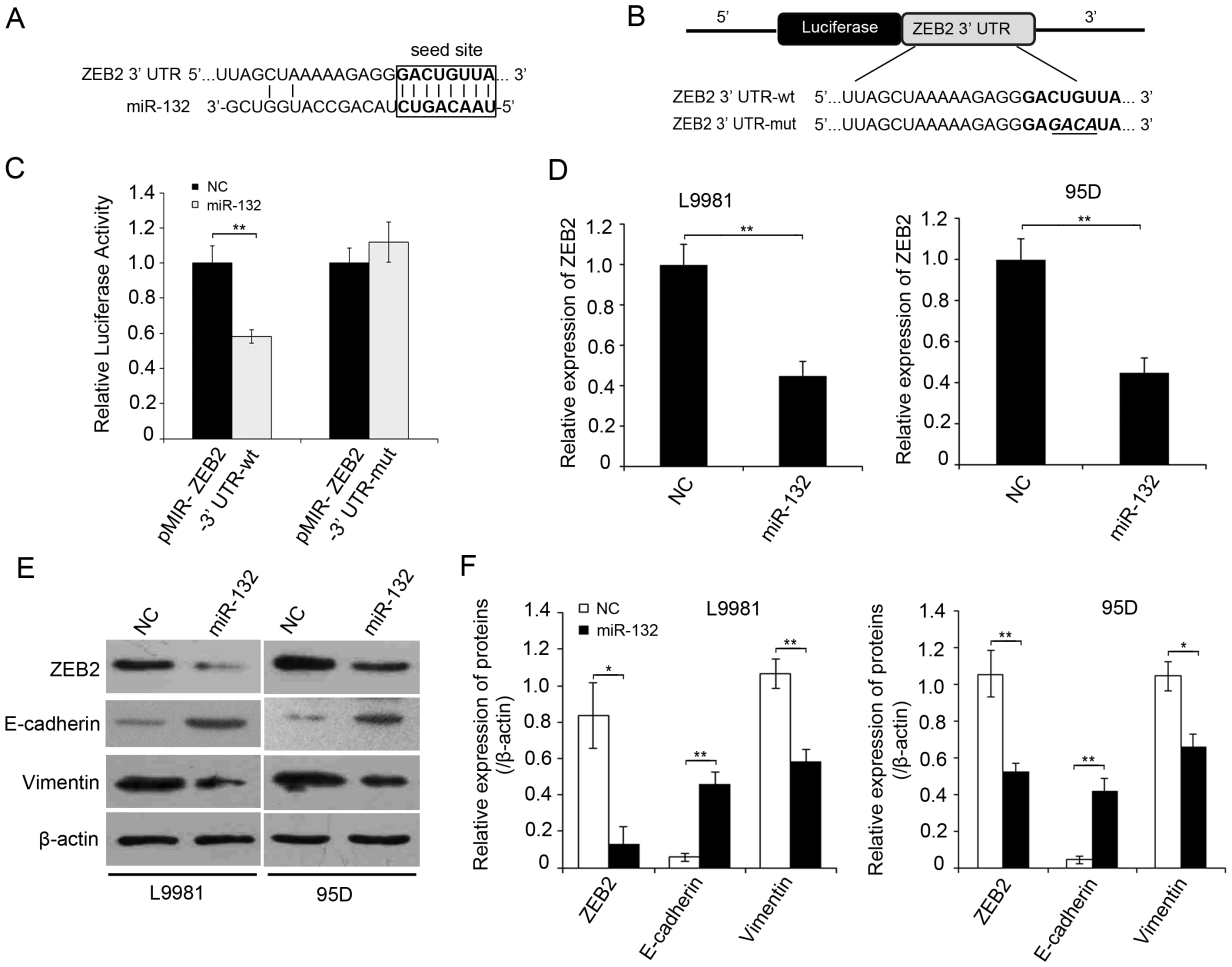
MiR-132 directly inhibits the expression of ZEB2 through its 3′UTR and regulates the EMT of NSCLC cells. (A) The miR-132 binding site predicted in the 3′UTR of ZEB2 mRNA. (B) Mutant was generated at the seed region of ZEB2 3′ UTR as indicated by the underline. A 3′ UTR fragment of ZEB2 mRNA containing wild-type or mutant of the miR-132 binding sequence was cloned into the downstream of the luciferase gene in pMIR vector. (C) L9981 cells were transfected with pMIR reporter vectors containing either wild-type or mutant ZEB2 3′UTR (indicated as pMIR-ZEB2-3′ UTR-wt and pMIR-ZEB2-3′ UTR-mut) with either pcDNA3.1 (indicated as NC) or pcDNA3.1-miR-132 vector (indicated as miR-132). Luciferase activity was determined 48 h after transfection. (D) ZEB2 mRNA was detected by qRT-PCR in cell lines transfected with pcDNA3.1 (indicated as NC) or pcDNA3.1-miR-132 vector (indicated as miR-132). (E, F) The protein levels of ZEB2, E-cadherin or vimentin was examined by Western blot in cells transfected with different plasmids. Figure F shows the relative gray values of each band (normalized to β-actin). Protein bands from three independent Western blot assays were quantified using Quantity One software (Bio-Rad, USA). Data are reported as mean ±SD (**P<0.01, Student’s *t*- test).

To detect the molecular mechanism by which miR-132 suppresses the metastasis of lung cancer cells, we predicted the putative target genes of miR-132 in human cells using the tool *miRanda*, *PicTar* and *TargetScans*. Among the predicted candidates, ZEB2 was of interest in this study, considering the crucial roles of ZEB2 in the development of human cancers [14], [15]. To test whether miR-132 directly targets ZEB2 (Figure 3A), the wild type or mutant 3′ UTR sequence of ZEB2 was cloned into pMIR reporter vector, respectively, as shown in Figure 3B. The luciferase activity of pMIR- ZEB2 3′ UTR-wt construct was significantly decreased upon the over-expression of miR-132 in NL9980 cells, whereas its mutant counterpart was not (Figure 3C). Of note, the mRNA or protein levels of ZEB2 in L9981 or 95D cells were dramatically reduced by miR-132, respectively (Figure 3D, 3E, and 3F). It is reported that ZEB2 is a vital EMT inducer through suppressing E-cadherin or inducing vimentin expression in human cancer [16], [19]. To further confirm that ZEB2 acts as a target of miR-132, we examine the effect of miR-132 on those two downstream effectors of ZEB2 by Western blot. As shown in Figure 3E and 3F, the EMT maker E-cadherin or vimentin was dramatically up-regulated or down-regulated upon the overexpression of miR-132 in both L9981 and 95D cells. Taken together, these data indicate that miR-132 directly inhibits ZEB2 expression *via* targeting its 3′ UTR and induces EMT of NSCLC cells.

### ZEB2 Contributes to miR-132- Suppressed Migration and Invasion of NSCLC Cells

**Figure 4:**
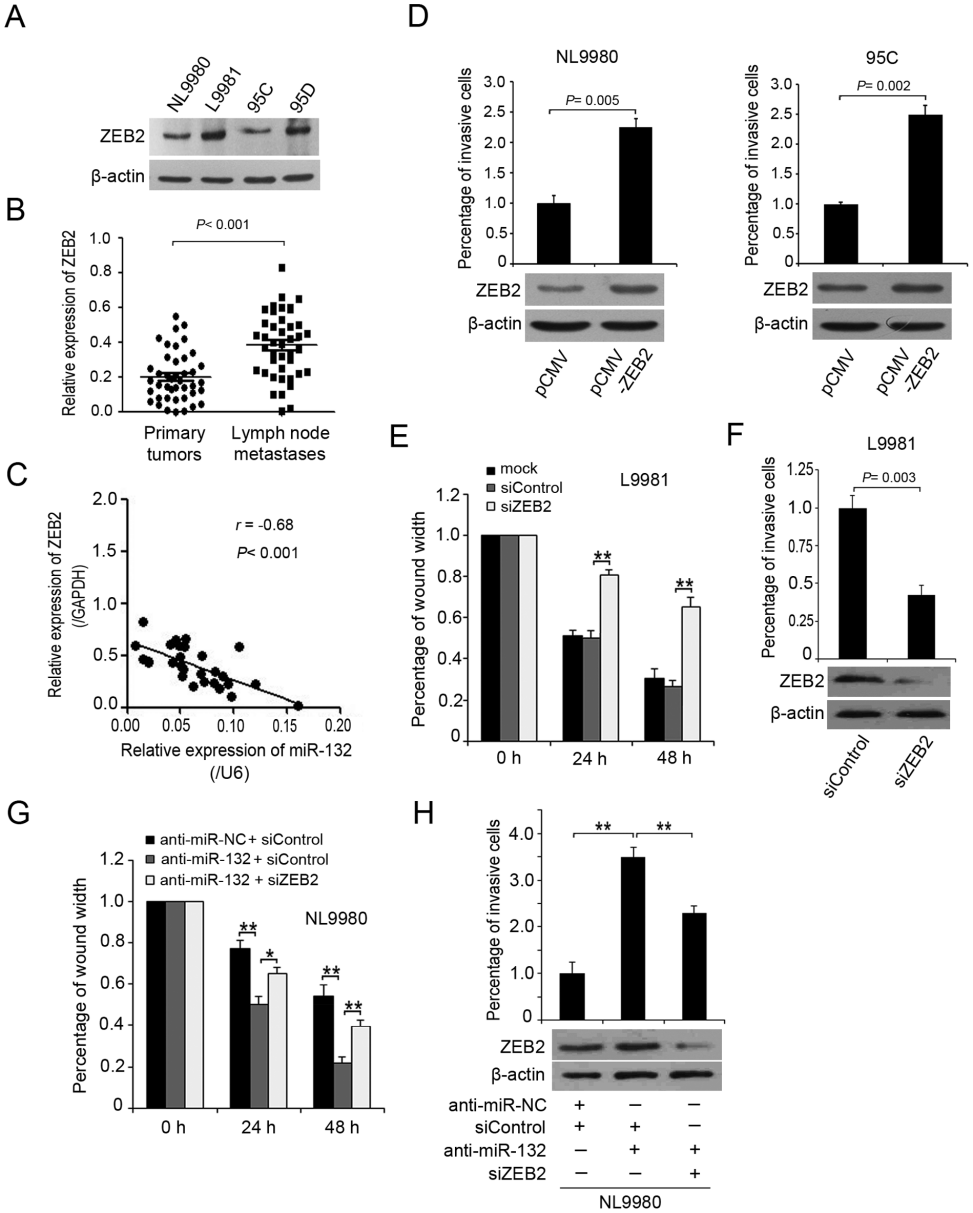
ZEB2 contributes to miR-132- suppressed migration and invasion of NSCLC cells. (A) The expression of ZEB2 was examined by Western blot in NSCLC cells. (B) The relative mRNA levels of ZEB2 were detected in 45 paired NSCLC primary tumor tissues and their lymph node metastasis counterparts. ZEB2 expression in those tissues was compared by way of Wilcoxon signed-rank test (***P<0.001, Student’s t- test). (C) Inverse correlation between miR-132 and ZEB2 expression in NSCLC tissues. ZEB2 expression was analyzed by qRT-PCR and normalized to GAPDH. The miR-132 expression was detected by qRT-PCR analysis and normalized to U6 expression. Statistical analysis was performed using Pearson’s correlation coefficient (r = −0.68, ***P<0.001). (D) The cell invasion was detected by Boyden chamber assay in NL9980 or 95C cells transfected with pCMV or pCMV-ZEB2 vectors, respectively. (E, F) The effect of ZEB2 knockdown on the cell migration or invasion was assessed by wound healing or Boyden chamber assay, respectively (**P<0.01, Student’s *t*- test). Additionally, the silencing efficiency of ZEB2 by siRNA was examined by Western blot. (G, H) The wound healing or Boyden chamber assay was used to detect the migration or invasion ability of NL9980 cells with different treatments, respectively (*P<0.05, **P<0.01, Student’s *t*- test). Additionally, the silencing efficiency of ZEB2 by siRNA was examined by Western blot.

Then, we examined the mRNA or protein expression of ZEB2 in several NSCLC cells, as well as 45 cases of primary lung cancer tissues and metastatic lymph node tissues. The data revealed that the protein level of ZEB2 in the highly metastatic L9981 or 95D cells was markedly up-regulated, compared to the poorly metastatic NL9980 or 95C cells, respectively (Figure 4A). Also, the same result about the mRNA levels of ZEB2 was observed in metastatic lymph node tissues, relative to the primary lung cancer tissues (Figure 4B). Of note, the expression of ZEB2 displayed a reverse correlation with miR-132 level in NSCLC tissues (Figure 4C). Next, we investigated whether ZEB2 is instrumental to the migration and invasion of NSCLC cells. Ectopic expression of ZEB2 in NL9980 or 95C cells significantly enhanced cell invasion (Figure 4D), however, silencing ZEB2 by siRNAs in L9981 cells resulted in decreased migration and invasion ability of the cells (Figure 4E, 4F), revealing its positive roles in the contribution of NSCLC cell migration and invasion. Meanwhile, the transfecting or silencing efficiency of ZEB2 in the cells was detected by Western blot (Figure 4D and 4F, *lower*). Then, we accessed whether the functional effect of miR-132 on NSCLC cells was dependent on ZEB2. As shown in Figure 4G and 4H, introduction of anti-miR-132 into NL9980 cells led to an increase of cell migration and invasion, whereas silence of ZEB2 by siRNAs partially abolished the enhancement. In parallel, the protein level of ZEB2 was confirmed by Western blot (Figure 4H, *lower*). Collectively, these results suggested that ZEB2 functions as a target of miR-132, responsible for miR-132-mediated regulation of the migration and invasion of NSCLC cells.

## Discussion

Our group has been focusing on the molecular mechanism of non-small cell lung cancer (NSCLC) development in recent years, especially devoting to the investigation of NSCLC metastasis. MiRNAs are involved in NSCLC pathogenesis and their expression profiles have been used to classify cancers [25], [26], [27]. Therefore, we expected that the dyregulation of metastasis-related microRNAs may facilitate the advanced progress of NSCLC. Miao LJ, *et*, *al.* found that miR-449c could inhibit the invasion of NSCLC cells by targeting c-Myc mRNA [28]. Also, Boning Liu, *et al.* reported that miR-26a may promote metastasis of lung cancer cells through activating AKT by targeting PTEN [29]. MiR-132, arising from the miR-212/132 cluster [30], has documented roles in the promotion of pancreatic cancer development *via* activating AKT signaling pathway [31]. However, the potential function of this microRNA in human NSCLC progression has few reports. In the present study, we are interested in the potential role of miR-132 in the metastasis of NSCLC cells.

In this study, we found that miR-132 was frequently down-regulated in highly metastatic NSCLC cell lines and tissue specimens. Thus, we supposed that miR-132 may be a novel tumor suppressor miRNA and its dyregulation may involve the advanced progress of human cancer. Besides, in the initial tumorigenesis, Shuyu Zhang and his colleague found that miR-132 exerted the low expression levels in primary pancreatic tumors and may be associated with the early development of human pancreatic cancer [13]. However, the potential role of miR-132 in the early progression of NSCLC still need to be further investigated. For the mechanism involving miR-132 down-regulation, Shuyu Zhang, *et al.* reported that the hyper-methylation in the promoter region was responsible for the reduced expression of miR-132 [13]. Accordingly, we speculated that this DNA modification might cause the alteration of miR-132 expression in human NSCLC.

Next, we investigated the function of miR-132 in NSCLC. Our data showed that the re-introduction of 132 dramatically repressed the cell migration and invasion of NSCLC cells *in vitro*. Therefore, our data suggested that the decreased expression of miR-132 may contribute to the metastasis of cancer cells and consequently facilitate the advanced development of human cancers like NSCLC. We further characterized ZEB2 as a functional target of miR-132 by luciferase reporter gene assays, RT-PCR and Western blot analysis, respectively. ZEB2/SIP1, as a member of the deltaEF-1 family of two-handed zinc-finger factors, is frequently expressed in a variety of human cancers, including pancreatic [31], breast [32], gastric [33], [34], renal [35], head and neck [36], glioma [37], hepatocellular [38], ovarian [39], squamous and non-small cell lung carcinomas [14], [15]. The important role of transcription factor ZEB2 has been strongly underlined in numerous papers, due to its function in inducing epithelial- mesenchymal transition (EMT) and facilitating the metastasis of cancer cells [37], [40], [41]. For instance, Nam EH, *et al*. reported that ZEB2 could induce EMT and invasion of human cancer cells *via* specifically repressing the expression of E-cateherin and up-regulating vimentin expression [42]. In addition, Cong N and his colleague found that down-regulated microRNA-200 family was able to promote EMT through wnt/β-catenin pathway by targeting E-cadherin repressors ZEB1/ZEB2 in gastric adenocarcinoma [33]. In the present study, we found that ZEB2 had a frequently high expression in metastatic NSCLC cells and clinical lymph node tissues. And ZEB2 was responsible for miR-132-modulated migration and invasion of NSCLC cells. Notably, we observed that E-cadherin or vimentin, the downstream effector of ZEB2, was also down-regulated or up-regulated by miR-132, indicating that miR-132 may exert functions in migration and invasion of NSCLC cells through modulating EMT. These *in vitro* data implied the necessary contribution of attenuated miR-132 in promoting cell metastasis in cancers. Recent studies revealed an mesenchymal- epithelial transition (MET) in metastases that allowed establishment of cancer cells in a remote site [43]. However, the potential role of miR-132 in this process still need to be further illustrated.

In summary, we investigated the role of miR-132 in NSCLC development. Our finding suggests that miR-132 may be a novel tumor suppressor miRNA. MiR-132 blocks the migration and invasion of NSCLC cells through targeting the EMT regulator ZEB2. Our data provide new insight into the mechanism responsible for the development of human NSCLC. Additionally, miR-132 may serve as a potential therapeutic candidate in the treatment of NSCLC.

## Supporting Information

Table S1 **Primers used in the paper were listed.**
(DOC)
