# Supplementary material for: MiR-132 Suppresses the Migration and Invasion of Lung Cancer Cells via Targeting the EMT Regulator ZEB2
Source: PLoS One. 2014 Mar 13;9(3):e91827. doi: 10.1371/journal.pone.0091827 (PMC3953608; doi:10.1371/journal.pone.0091827)
Supplement: Table S1 — Primers used in the paper were listed. (DOC) [file pone.0091827.s001.doc]

**Table S1.** Primers used in the paper were listed.

| **Gene** | **Primer** | **Sequence (5′-3′)** |
| --- | --- | --- |
| **Primers for qRT-PCR** | |  |
| U6 | forward | CTCGCTTCGGCAGCACA |
|  | reverse | AACGCTTCACGAATTTGCGT |
| ZEB2 | forward | ACCAGCGGAAACAAGGAT |
|  | reverse | TTTATGTCGCAGAAGGGAAC |
| GAPDH | forward | CATCACCATCTTCCAGGAGCG |
|  | reverse | TGACCTTGCCCACAGCCTTG |
| **Primers for miR-132 sequence** | | |
| miR-132 | forward | TGGATCCCCCCCAGTCCCCGTCCCTCAG |
|  | reverse | TGAATTCGGATACCTTGGCCGGGAGGAC |
| **Primers for ZEB2 3’UTR** | | |
| ZEB2 3’UTR-wt | forward | CATTTTATATTTCCTAATTT |
|  | reverse | CATAGGTATGGAATTTTAAAAAACA |
| ZEB2 3’UTR-mut | forward | TATGTCTCCCTCTTTTTAGCTAA |
|  | reverse | TAAAAAGAGGGAGACATAAATACAATGTATGATA |
| **Primers for ZEB2 CDS** | | |
| ZEB2-CDS | forward | CGATATCATGAAGCAGCCGATCATG |
|  | reverse | CGTCGACTTACATGCCATCTTCCATATTGT |
|  |  |  |
